# Tislelizumab for squamous lung cancer combined with basal cell carcinoma of the skin: A case report

**DOI:** 10.1097/MD.0000000000033788

**Published:** 2023-05-12

**Authors:** Ming-Jun Wu, Yu-Chun Chen, Xiao-Li Cui, Qian Yang, Qing-Liang Xue

**Affiliations:** a Respiratory, The 940th Hospital of Joint Logistics Support Force of Chinese People’s Liberation Army, Lanzhou, Gansu Province, China; b Respiratory, The Clinical Medical College of Ningxia Medical University, Yinchuan, Ningxia Hui Autonomous Region, China.

**Keywords:** basal cell carcinoma of the skin, lung squamous cell carcinoma, PD-1 inhibitors, tislelizumab

## Abstract

Surgery is the preferred treatment for basal cell carcinoma (BCC), locally advanced or metastatic BCC, radiation therapy or systemic therapy can be considered. Programmed death receptor 1 (PD-1) inhibitors are rarely used to treat cutaneous BCC. In the present case, we found that tislelizumab, a PD-1 immunosuppressant, had a positive effect on BCC.

**Patient concerns:**

A 74-year-old male patient presented with a mass in the left back in October 2021, which was surgically removed and diagnosed as BCC. The patient was diagnosed with squamous lung cancer after presenting with a cough and coughing up a small amount of white, sticky sputum in December 2021.

**Diagnosis:**

BCC and squamous lung cancer.

**Interventions:**

Docetaxel + nedaplatin systemic chemotherapy combined with tislelizumab immunotherapy.

**Outcomes:**

Both BCC and squamous lung cancer were significantly reduced in size.

**Conclusion:**

After 2 cycles of immunotherapy with tislelizumab, the lung tumor shrank, the back mass disappeared, and the wound healed.

## 1. Introduction

Basal cell carcinoma (BCC) is the most common malignant tumor of the skin, with poor growth, less metastasis, and favorable prognosis. Surgical resection is the preferred treatment, with a recurrence rate of 2% to 8% 5 years after the operation. Radiotherapy or systemic treatment can be considered for locally advanced or metastatic BCCs. However, programmed death receptor 1 (PD-1) inhibitors have rarely been used for the treatment of BCC of the skin. In the present case, we found that tislelizumab, a PD-1 inhibitor, was effective in treating both squamous cell carcinoma of the lung and BCC of the skin. In this paper, we report this case and review the literature to provide a reference for the treatment of advanced BCC of the skin.

## 2. Case presentation

The patient was a 74-year-old male. In October 2021, a subcutaneous mass appeared in the left scapular region of his back, which was hard and poorly mobile and gradually increased in size. When the diameter was approximately 3cm, it broke down with pain. The tumor was removed at a local hospital. The pathological diagnosis was (back) BCC. After surgery, the incision did not heal, and the abscess repeatedly broke. There was no significant improvement after reoperation. In December 2021, he developed a cough and coughed a small amount of white, sticky phlegm. Chest computed tomography (CT) showed an irregular mass in the left upper lung, which was considered lung cancer. Bronchoscopy revealed that polypoid neoplasms completely obstructed the left upper lobe bronchus, and lesion invaded the left lower lobe bronchus. The pathological diagnosis was squamous cell carcinoma (the left upper lobe bronchus), grade II; epidermal growth factor receptor, anaplastic lymphoma kinase, proto-oncogene 1 receptor tyrosine kinase, and other driving genes were negative; The programmed death ligand 1 (PD-L1) (SP263) tumor cell proportion score was positive for tumor cells (≈60%). Docetaxel (120 mg D1) and nedaplatin (100 mg D1) were administered for 2 cycles of systemic chemotherapy. A repeat chest CT scan showed no reduction in the left upper lung mass and no significant improvement in the left scapular area wound. A new subcutaneous mass appeared, and a puncture biopsy was performed to diagnose BCC of the skin, which was considered a metastatic lesion. The treatment regimen was adjusted to docetaxel (120 mg D1) + nedaplatin (100 mg D1) systemic chemotherapy, combined with tislelizumab (200 mg D1) immunotherapy. After 2 cycles, the wound in the left scapular region healed, and the new subcutaneous swelling disappeared. Later, the treatment was interrupted for 3 months for personal reasons, and a subcutaneous tumor reappeared on the patient left shoulder, which disappeared after 1 cycle of immunotherapy. At present, the patient has received 5 cycles of systemic chemotherapy and 3 cycles of tislelizumab immunotherapy. The lung tumor shrank, the clinical symptoms decreased, and no new subcutaneous masses appeared. The patient remains under follow-up.

**Figure 1.**
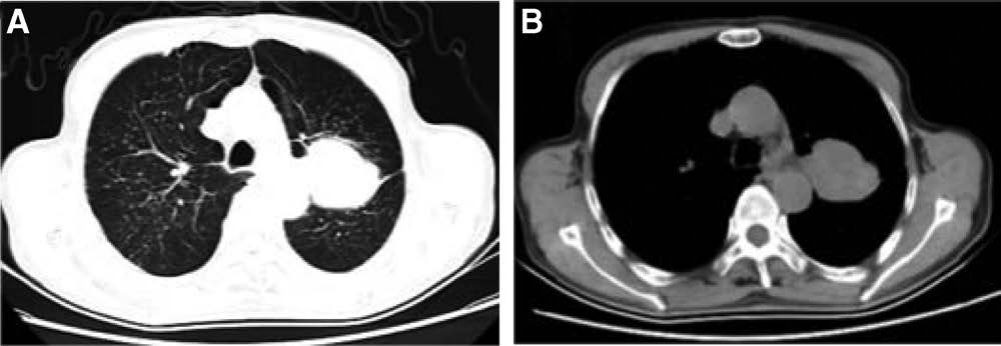
(A and B). Chest CT revealed an irregular mass in the upper lobe of the left lung. CT = computed tomography.

**Figure 2.**
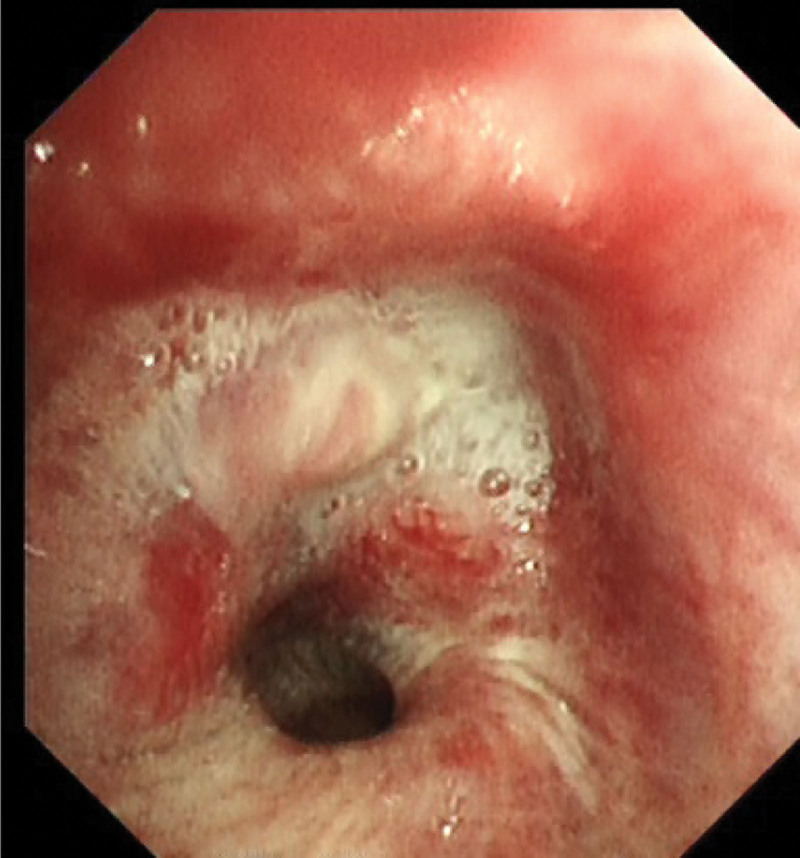
Bronchoscopy revealed polypoid neoplasms completely obstructed the left upper lobe bronchus.

**Figure 3.**
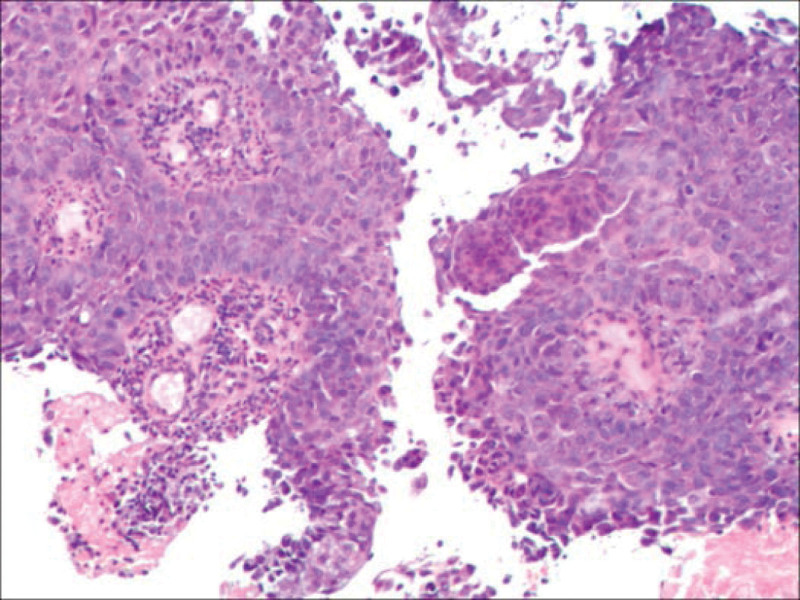
Biopsy pathology of the left upper lung showed that the cancerous tissue was arranged in a nested pattern with large, deep-stained nuclei. Diagnosis: Grade II Squamous cell carcinoma.

**Figure 4.**
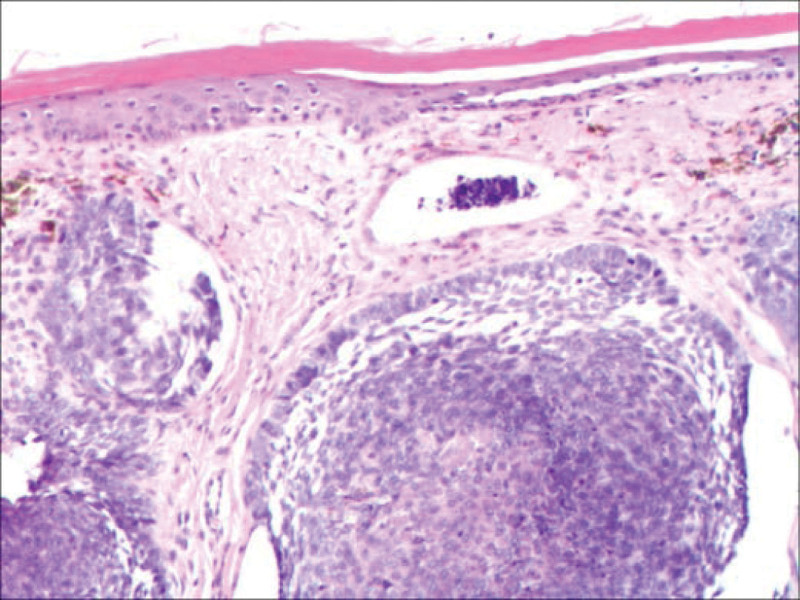
Biopsy pathology of the back mass showed that the tumor cells were arranged in nests of different sizes and shapes, in which fibrous connective tissue could be observed, and the peripheral cells of the cell mass were arranged in a palisading pattern. Diagnosis: Basal cell carcinoma.

Chest CT (Fig. 1A and B) revealed an irregular mass in the upper lobe of the left lung. Bronchoscopy (Fig. 2): Polypoid neoplasms completely obstructed the left upper lobe bronchus, and lesion invaded the left lower lobe bronchus. Biopsy pathology of the left upper lung (Fig. 3): The cancerous tissue was arranged in a nested pattern with large, deep-stained nuclei. Diagnosis: Grade II Squamous cell carcinoma epidermal growth factor receptor, anaplastic lymphoma kinase, proto-oncogene 1 receptor tyrosine kinase, and other driving genes were negative. PD-L1 (SP263) tumor cell proportion score was positive for tumor cells at approximately 60%. Biopsy pathology of the back mass (Fig. 4): The tumor cells were arranged in nests of different sizes and shapes, in which fibrous connective tissue could be observed, and the peripheral cells of the cell mass were arranged in a palisading pattern. Diagnosis: BCC. Lung tumor index: carcinoembryonic antigen 17.17 ng/mL, cytokeratin 19 fragments 5.46 ng/mL, squamous cell carcinoma antigen 2.60 ng/mL.

There was no history of disease. There was no history of surgery or trauma. There was no history of food or drug allergy, and no relevant personal or family history.

## 3. Discussion

In recent years, immunotherapy has made historic breakthroughs in the treatment of a variety of tumors, among which PD-1/PD-L1 inhibitors have been applied in a variety of malignant tumors.^[1]^ The binding of PD-1 and PD-L1 inhibits the proliferation of T cells and affects their survival of T cells by suppressing the production of interferon-γ, tumor necrosis factor-α, and interleukin-2. Thus the anti-tumor immune response is inhibited by making tumor cells unrecognizable to immune cells.^[2]^ PD-1/PD-L1 targeted inhibitors include monoclonal antibodies, polypeptide inhibitors, small molecule inhibitors, and anti-cancer vaccines. They can block PD-1/PD-L1 interaction, activate T cells, and restore their anti-tumor immune response.^[3]^ Tislelizumab^[4,5]^ is a humanized monoclonal antibody against PD-1(IgG4 variant) that was independently developed in China. Its antibody structure differ from that of the traditional PD-1 antibody. Its fragment crytallizable segment has been genetically engineered to have a lower affinity for fragment crytallizable-γ receptor 1, effectively avoiding the antibody-dependent phagocytosis effect, and recognizes the key epitopes Gln75, Thr76, Asp77, and Arg86 on PD-1 with higher specificity and affinity for PD-1 expressed on the surfaces of CD4 + and CD8 + T cells, B lymphocytes, dendritic cells, and natural killer cells. It completely blocks the interaction between PD-1 and PD-L1 expressed on the surface of malignant tumor cells, tumor-infiltrating lymphocytes, antigen-presenting cells, and tumor-infiltrating dendritic cells, which restores the body immune system to monitor and kill tumors^[6,7]^; At present, tislelizumab has been approved in China for the treatment of classical Hodgkin lymphoma, urothelial carcinoma, non-small cell lung cancer (NSCLC), hepatocellular carcinoma, esophageal squamous cell carcinoma, and gastric/gastroesophageal junction cancer. In recent years, some researchers have tried to use tislelizumab for refractory renal cell carcinoma, cholangiocarcinoma, and serous endometrial carcinoma, all of which have achieved good therapeutic effects.^[5]^

Lung cancer is a malignant tumor with the highest morbidity and mortality worldwide.^[8]^ Lung squamous cell carcinoma is one of the most common pathological types of NSCLC.^[9]^ Local surgical resection can be used for treatment in the early stages, with a 5-year survival rate of approximately 40% to 50%. Targeted drug treatment can be selected for advanced patients with positive driver genes, whereas systemic chemotherapy combined with immunotherapy is the main tool for advanced patients with negative driver genes. The Chinese Society of Clinical Oncology has recommended “paclitaxel/albumin paclitaxel + platinum, combined with pembrolizumab/tislelizumab" as the first-line therapeutic regimen.

BCC is the most common malignancy worldwide.^[10]^ Surgery is the main treatment modality, but superficial BCC can also be treated with non-surgical procedures, such as photodynamic or topical medications.^[11,12]^ Hedgehog pathway inhibitors (HHI) are approved for the treatment of metastatic or locally advanced BCC, but most patients experience disease progression when treated with HHI.^[13,14]^ Adverse reactions caused by HHI also limit its use.^[15]^ Studies have found that^[16,17]^ BCC usually has a high tendency of gene mutations. PD-1 and PD-L1 were significantly expressed in patients with BCC. PD-1 inhibitors have potential efficacy in advanced patients who still progress after HHI treatment, which is regarded as salvage therapy.^[18]^ Cemiplimab, a PD-1 antibody, was approved by the US Food and Drug Administration in February 2021 for patients with locally advanced BCC^[19,20]^ and has shown promising antitumor activity and safety. It has also been reported^[21,22]^ that several patients with BCC had tumor regression after treatment with the PD-1 inhibitor nivolumab.

## 4. Conclusion

**Figure 5.**
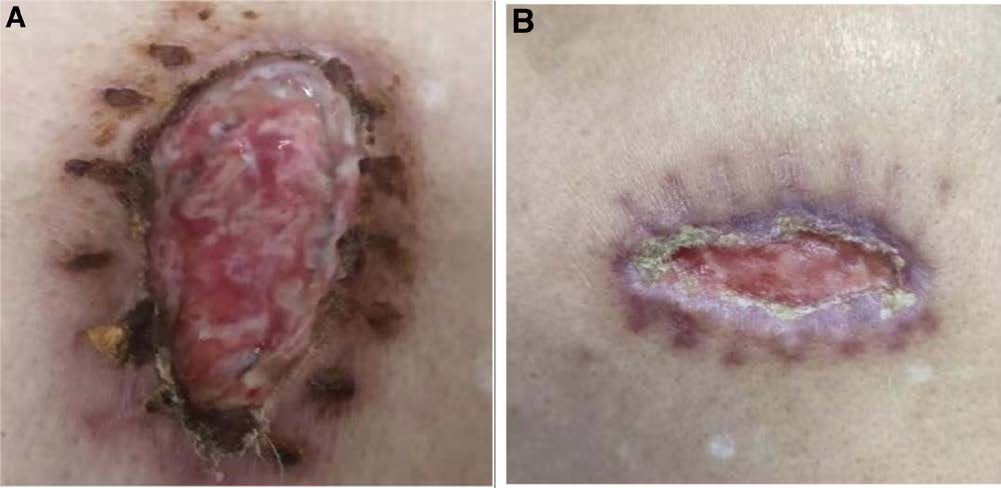
(A) The wound did not heal after surgery. (B) The wound healed after 2 cycles of immunotherapy.

Tislelizumab, a PD-1 immunosuppressant, has been widely used for the treatment of NSCLC with good results. However, it has not been used for the treatment of skin BCC, and there are no relevant reports. In the present case, the patient was diagnosed with skin BCC and underwent surgical resection, however, the postoperative wound did not heal (Fig. 5A). Two months later, the patient was diagnosed with squamous cell carcinoma of the lung. After 2 cycles of systemic chemotherapy with docetaxel + nedaplatin, the wound did not significantly improve. A new subcutaneous mass appeared next to the wound, which was confirmed to be BCC of the skin by puncture biopsy, and metastasis was considered. Simultaneously, the lung tumor did not shrink significantly after 2 cycles of chemotherapy. The treatment regimen was adjusted to docetaxel plus nedaplatin systemic chemotherapy combined with tislelizumab immunotherapy. After 2 cycles of treatment, the wound healed (Fig. 5B), and the new swelling disappeared. Subsequently, the treatment was interrupted for 3 months for personal reasons, and a subcutaneous tumor appeared again on the left shoulder of the patient. After 1 cycle of immunotherapy, the tumor had disappeared. In conclusion, the present case revealed that tislelizumab was effective for the treatment of skin BCC in this case, and the effect was significant. We look forward to more studies in the future to confirm the effectiveness of tislelizumab in the treatment of BCC of the skin, thus providing new ideas for the treatment of advanced and metastatic BCC.

## Author contributions

**Conceptualization:** Ming-Jun Wu, Yu-Chun Chen.

**Data curation:** Qing-Liang Xue.

**Formal analysis:** Qing-Liang Xue.

**Methodology:** Qing-Liang Xue.

**Resources:** Xiao-Li Cui, Qian Yang.

**Writing – original draft:** Ming-Jun Wu, Yu-Chun Chen.
